# Supplementary material for: Population Structure of Modern Winter Wheat Accessions from Central Asia
Source: Plants (Basel). 2023 Jun 6;12(12):2233. doi: 10.3390/plants12122233 (PMC10305188; doi:10.3390/plants12122233)

Genome A
667 samples
(10 groups)
4129 SNPs

Principal Coordinates Analysis (PCoA)

Pairwise Population Matrix of **Nei Unbiased Genetic Distance**

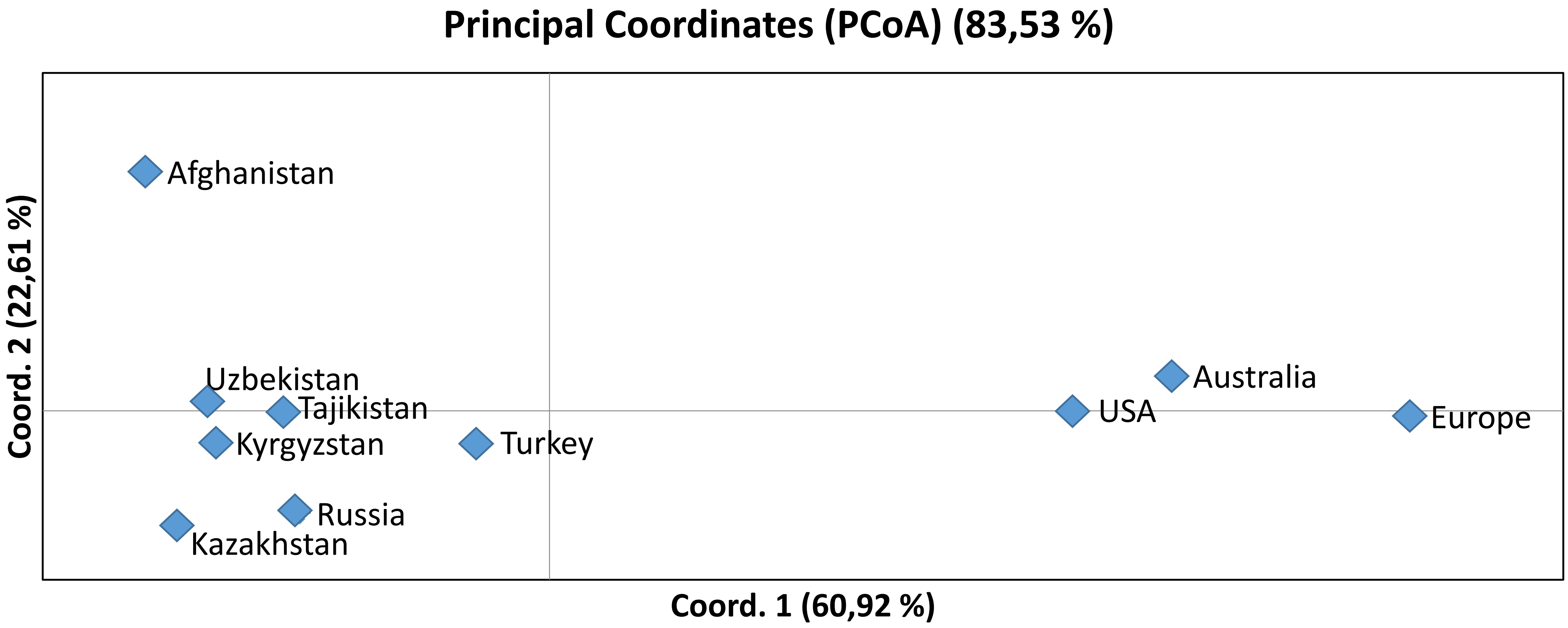

Genome B
667 samples
(10 groups)
5080 SNPs

Principal Coordinates Analysis (PCoA)

Pairwise Population Matrix of **Nei Unbiased Genetic Distance**

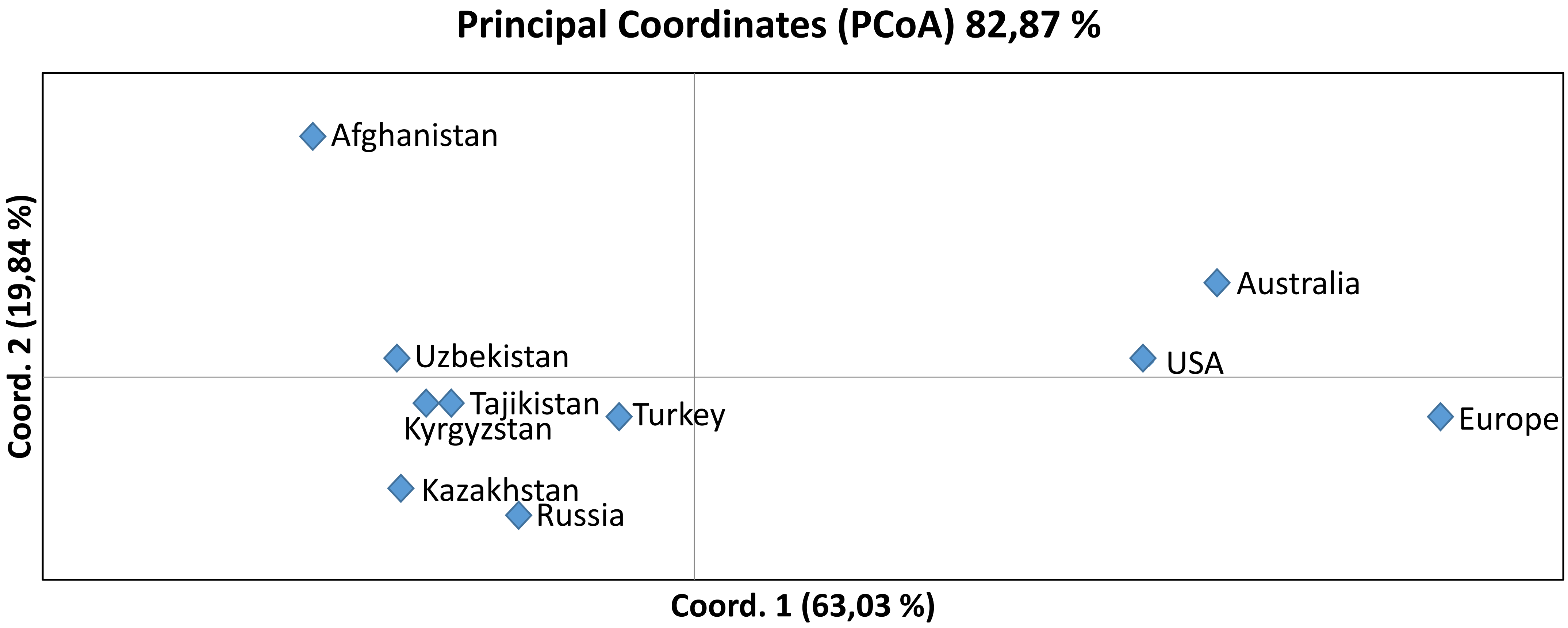

Genome D
667 samples
(10 groups)
1526 SNPs

Principal Coordinates Analysis (PCoA)

Pairwise Population Matrix of **Nei Unbiased Genetic Distance**

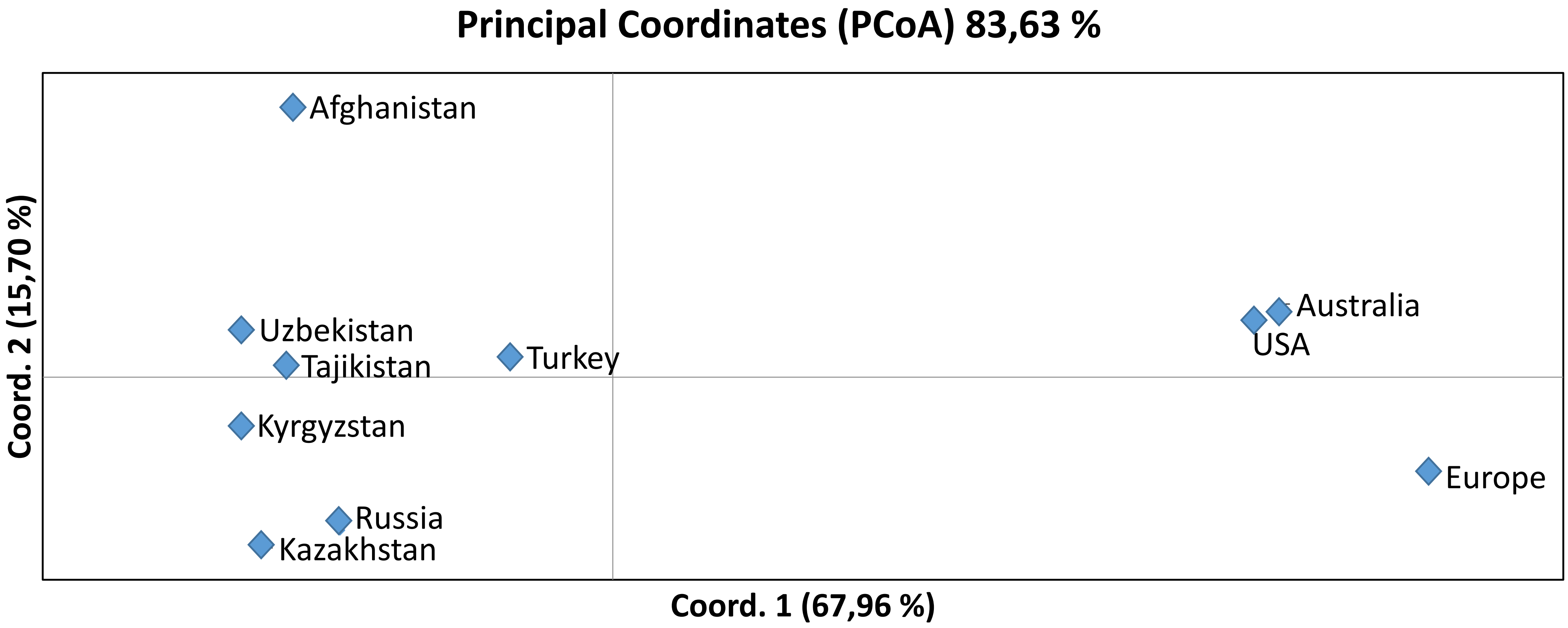

1A chromosome
667 samples
(10 groups)
633 SNPs

Principal Coordinates Analysis (PCoA)

Pairwise Population Matrix of **Nei Unbiased Genetic Distance**

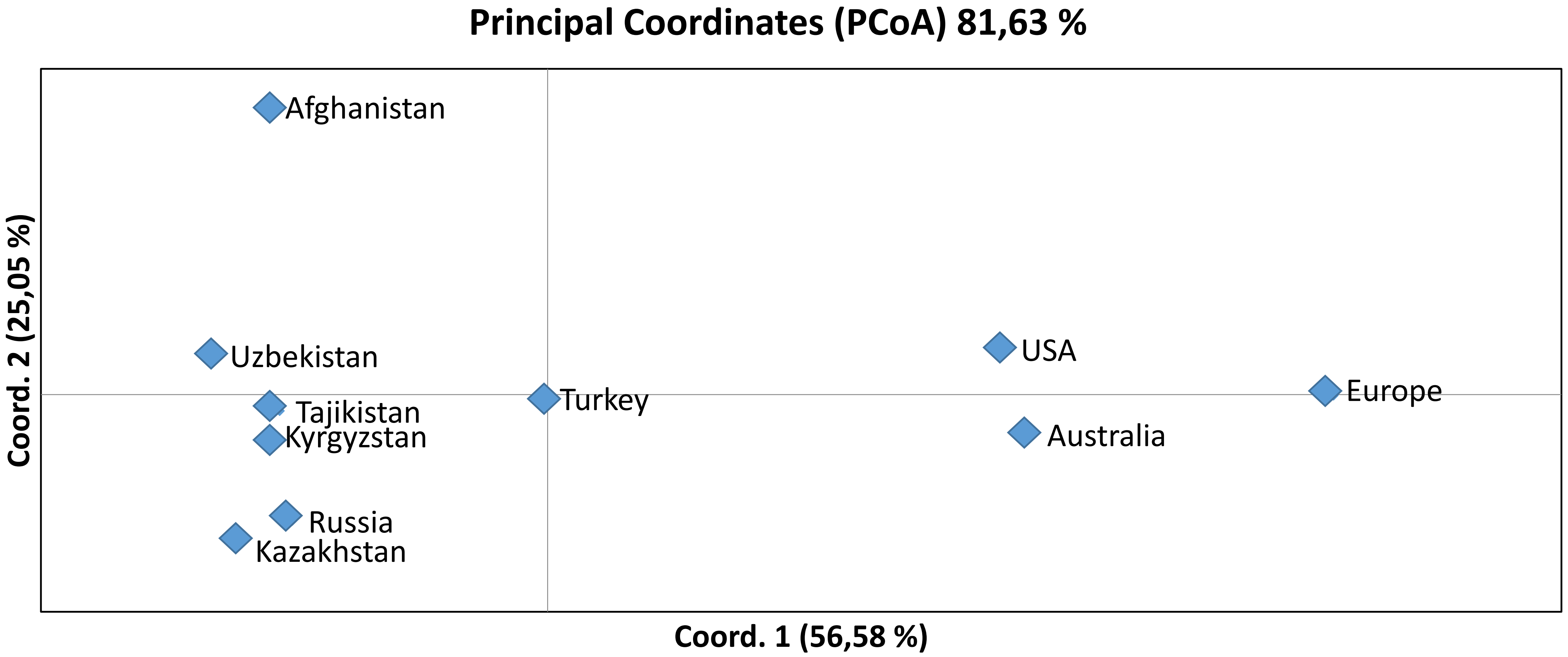

1B chromosome
667 samples
(10 groups)
975 SNPs

Principal Coordinates Analysis (PCoA)

Pairwise Population Matrix of **Nei Unbiased Genetic Distance**

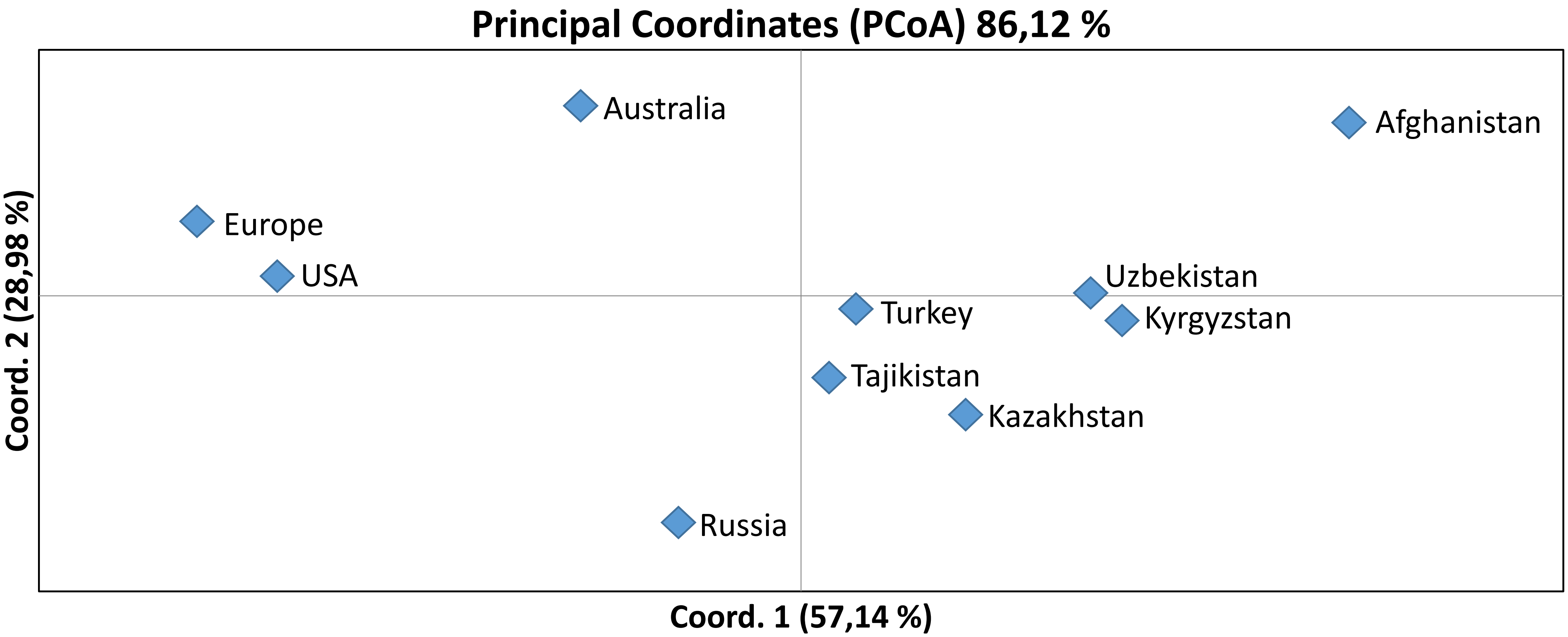

1D chromosome
667 samples
(10 groups)
328 SNPs

Principal Coordinates Analysis (PCoA)

Pairwise Population Matrix of **Nei Unbiased Genetic Distance**

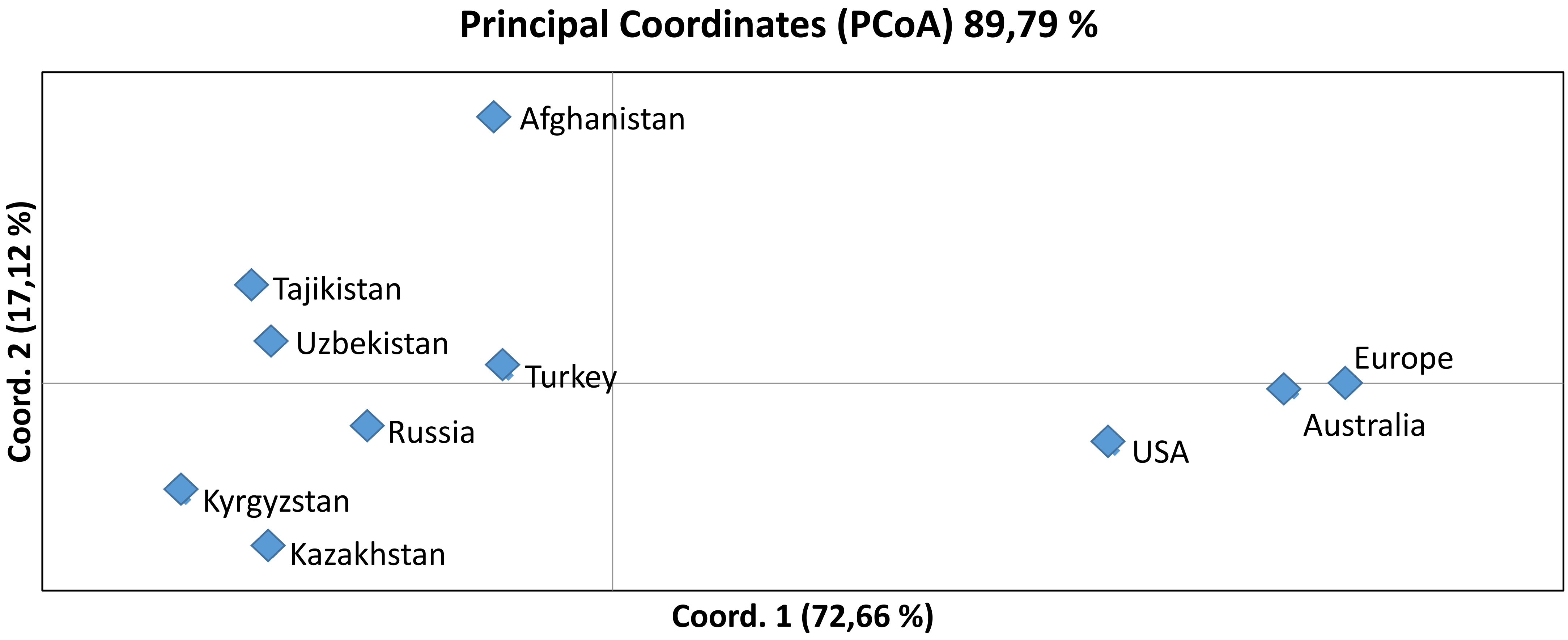

2A chromosome
667 samples
(10 groups)
738 SNPs

Principal Coordinates Analysis (PCoA)

Pairwise Population Matrix of **Nei Unbiased Genetic Distance**

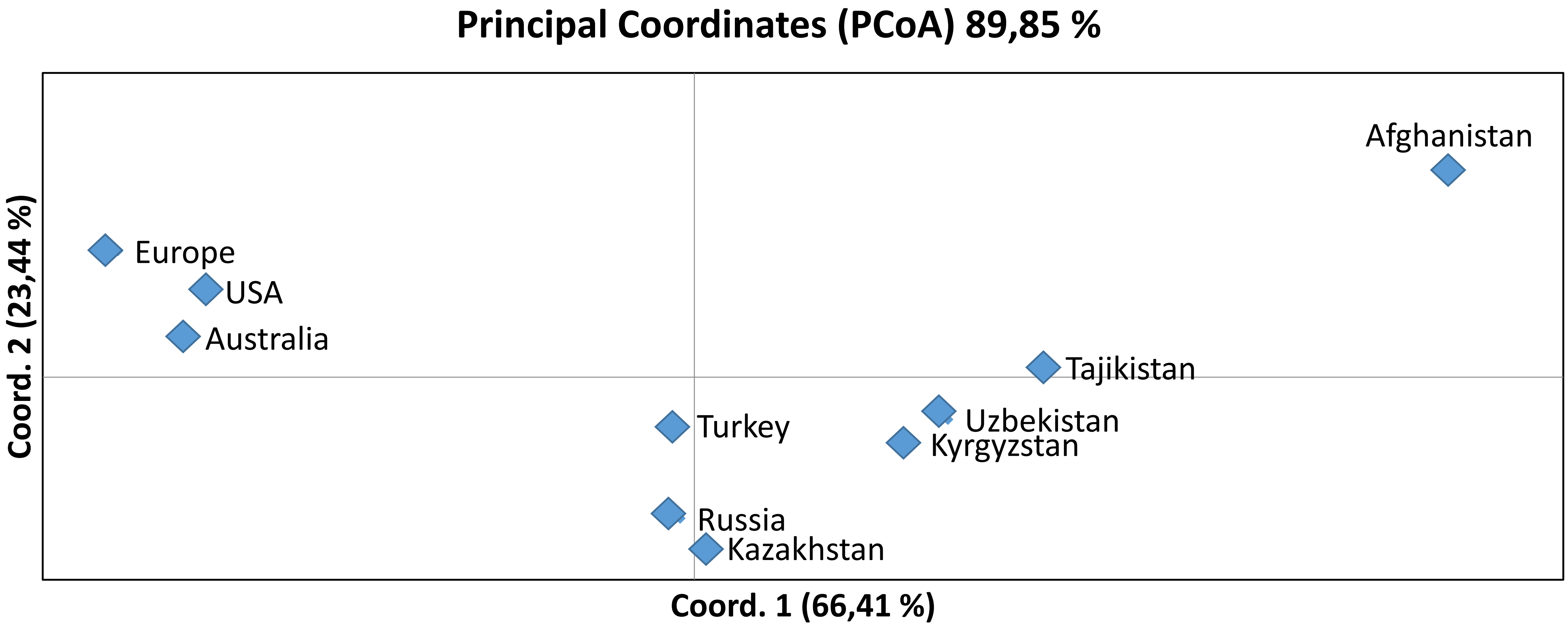

2B chromosome
667 samples
(10 groups)
849 SNPs

Principal Coordinates Analysis (PCoA)

Pairwise Population Matrix of **Nei Unbiased Genetic Distance**

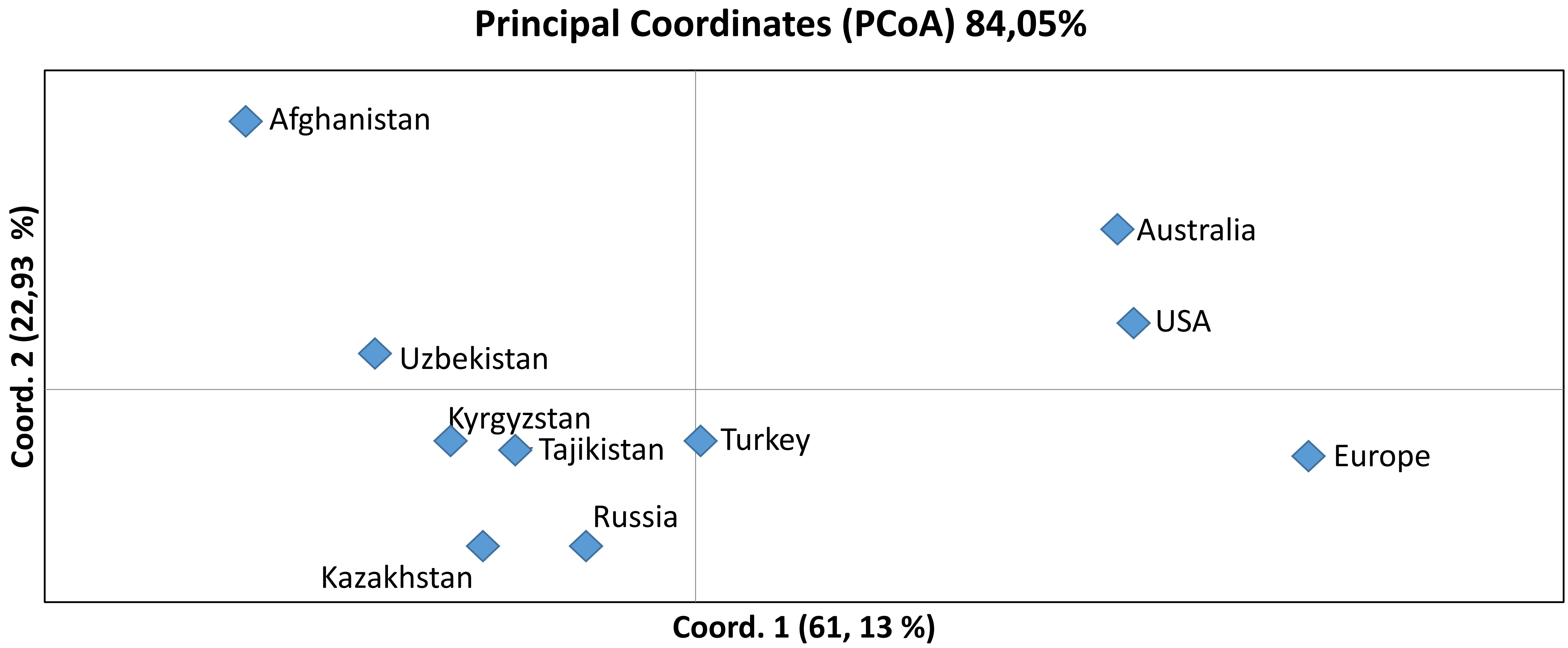

2D chromosome
667 samples
(10 groups)
368 SNPs

Principal Coordinates Analysis (PCoA)

Pairwise Population Matrix of **Nei Unbiased Genetic Distance**

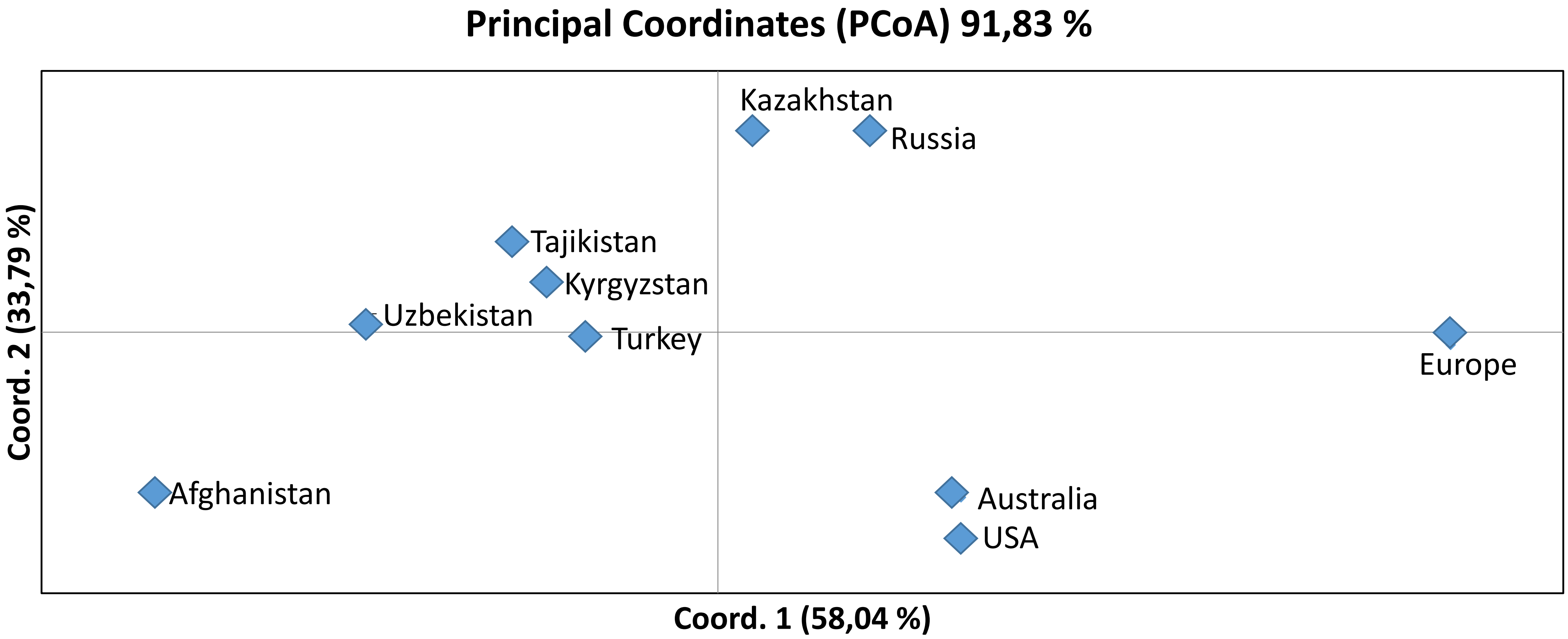

3A chromosome
667 samples
(10 groups)
525 SNPs

Principal Coordinates Analysis (PCoA)

Pairwise Population Matrix of **Nei Unbiased Genetic Distance**

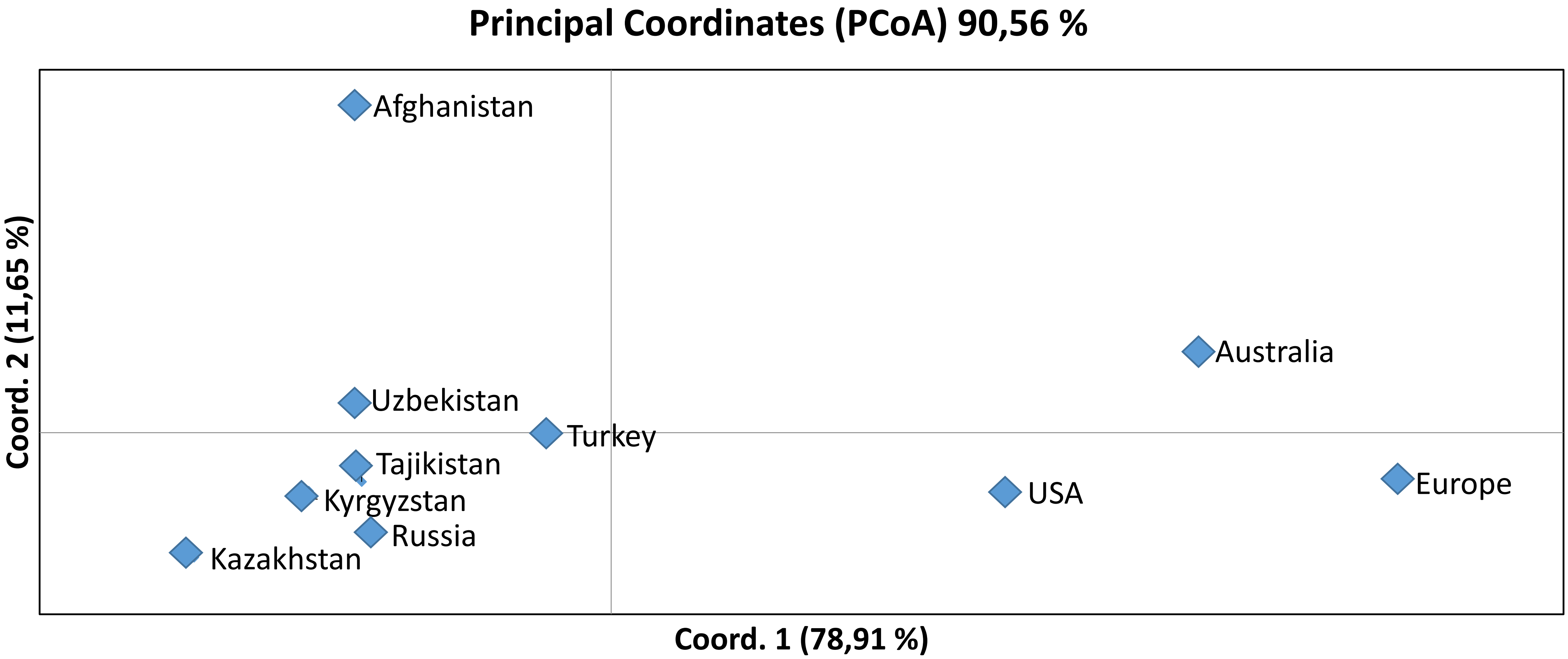

3B chromosome
667 samples
(10 groups)
747 SNPs

Principal Coordinates Analysis (PCoA)

Pairwise Population Matrix of **Nei Unbiased Genetic Distance**

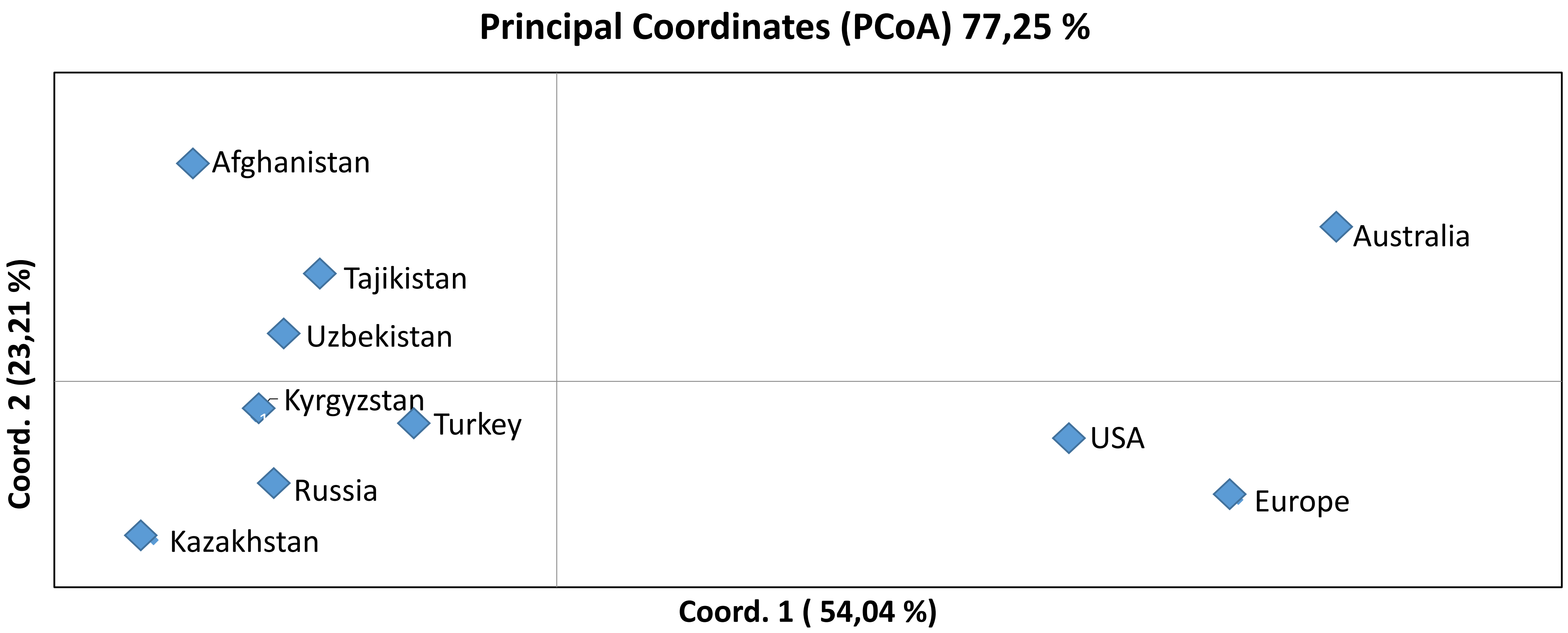

3D chromosome
667 samples
(10 groups)
191 SNPs

Principal Coordinates Analysis (PCoA)

Pairwise Population Matrix of **Nei Unbiased Genetic Distance**

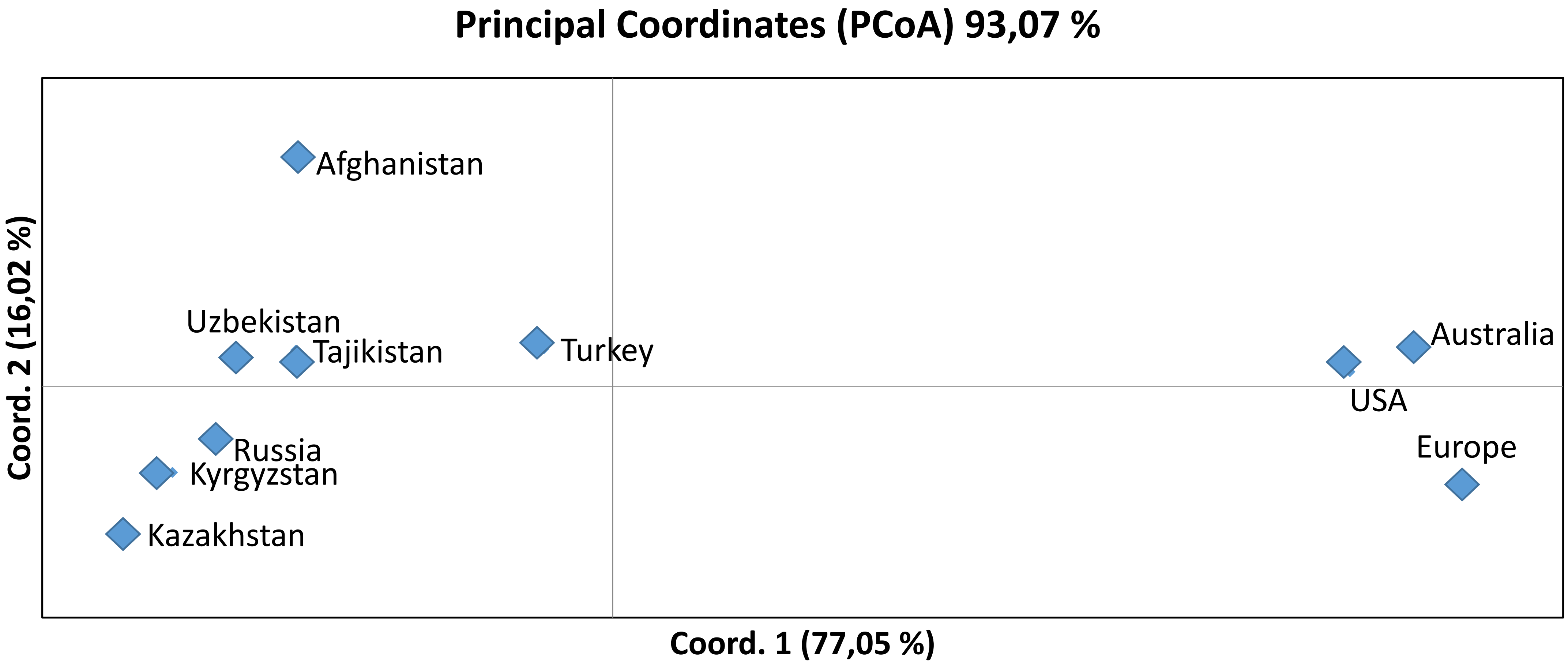

4A chromosome
667 samples
(10 groups)
348 SNPs

Principal Coordinates Analysis (PCoA)

Pairwise Population Matrix of **Nei Unbiased Genetic Distance**

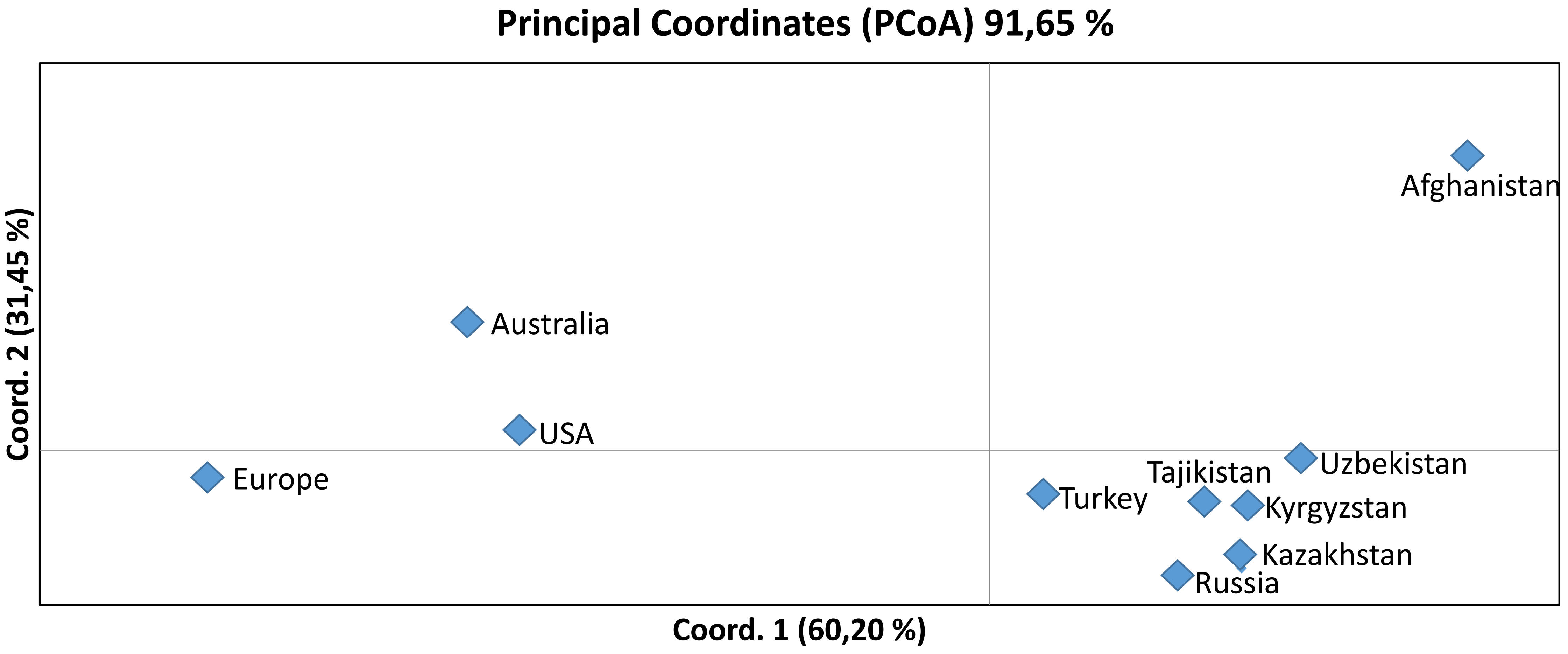

4B chromosome
667 samples
(10 groups)
429 SNPs

Principal Coordinates Analysis (PCoA)

Pairwise Population Matrix of **Nei Unbiased Genetic Distance**

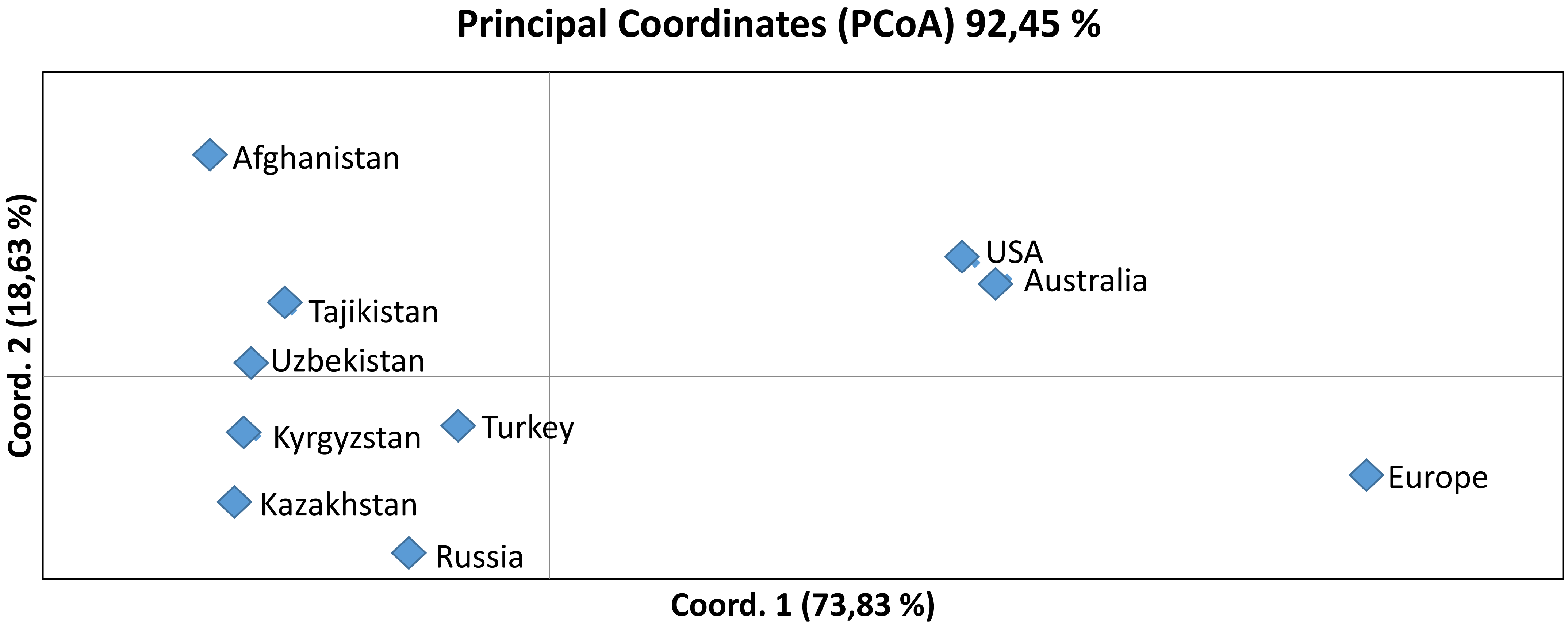

4D chromosome
667 samples
(10 groups)
429 SNPs

Principal Coordinates Analysis (PCoA)

Pairwise Population Matrix of **Nei Unbiased Genetic Distance**

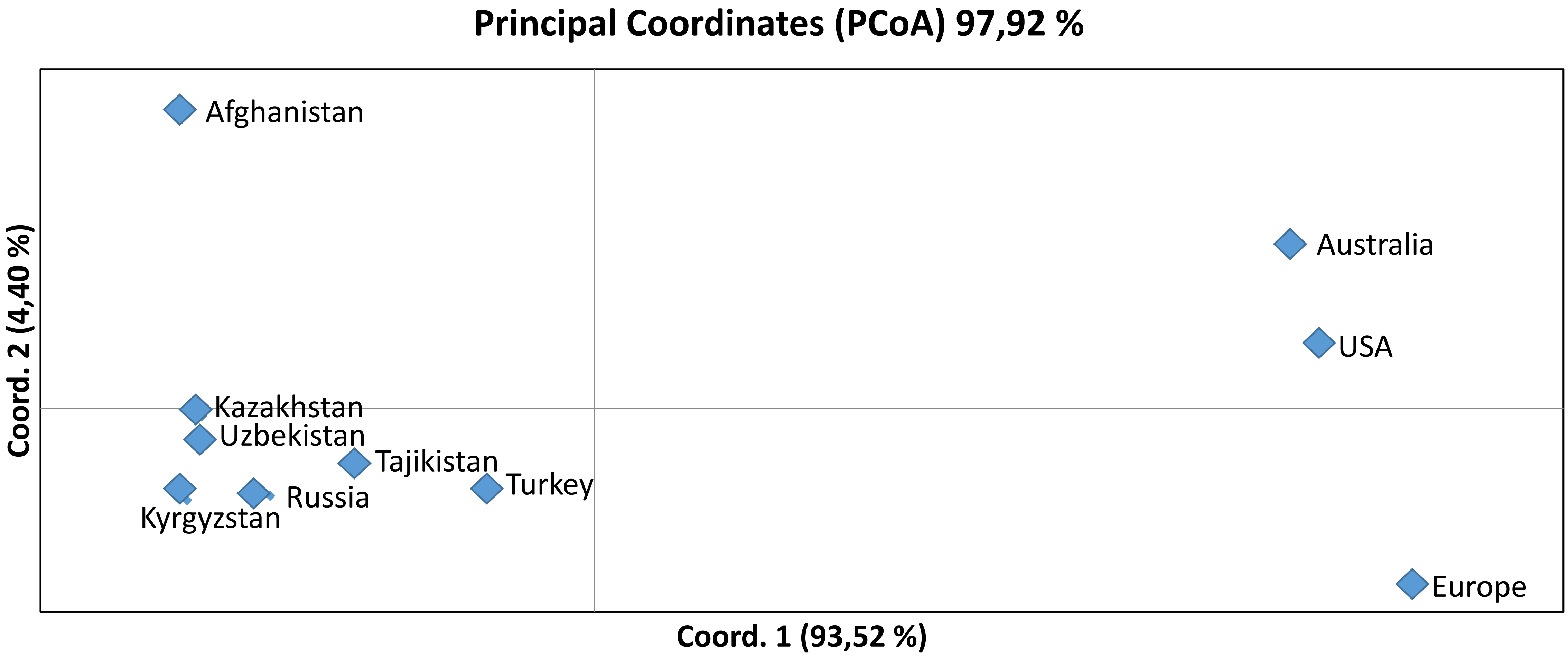

5A chromosome
667 samples
(10 groups)
688 SNPs

Principal Coordinates Analysis (PCoA)

Pairwise Population Matrix of **Nei Unbiased Genetic Distance**

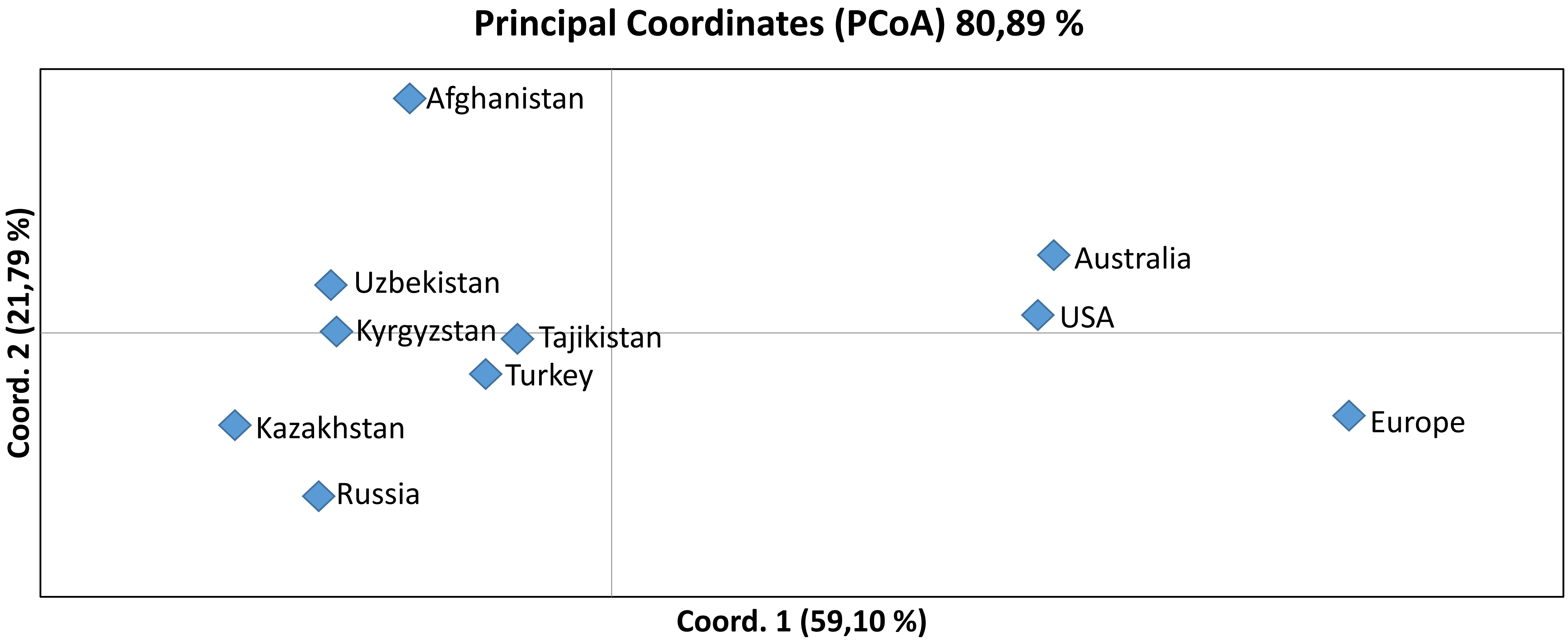

5B chromosome
667 samples
(10 groups)
870 SNPs

Principal Coordinates Analysis (PCoA)

Pairwise Population Matrix of **Nei Unbiased Genetic Distance**

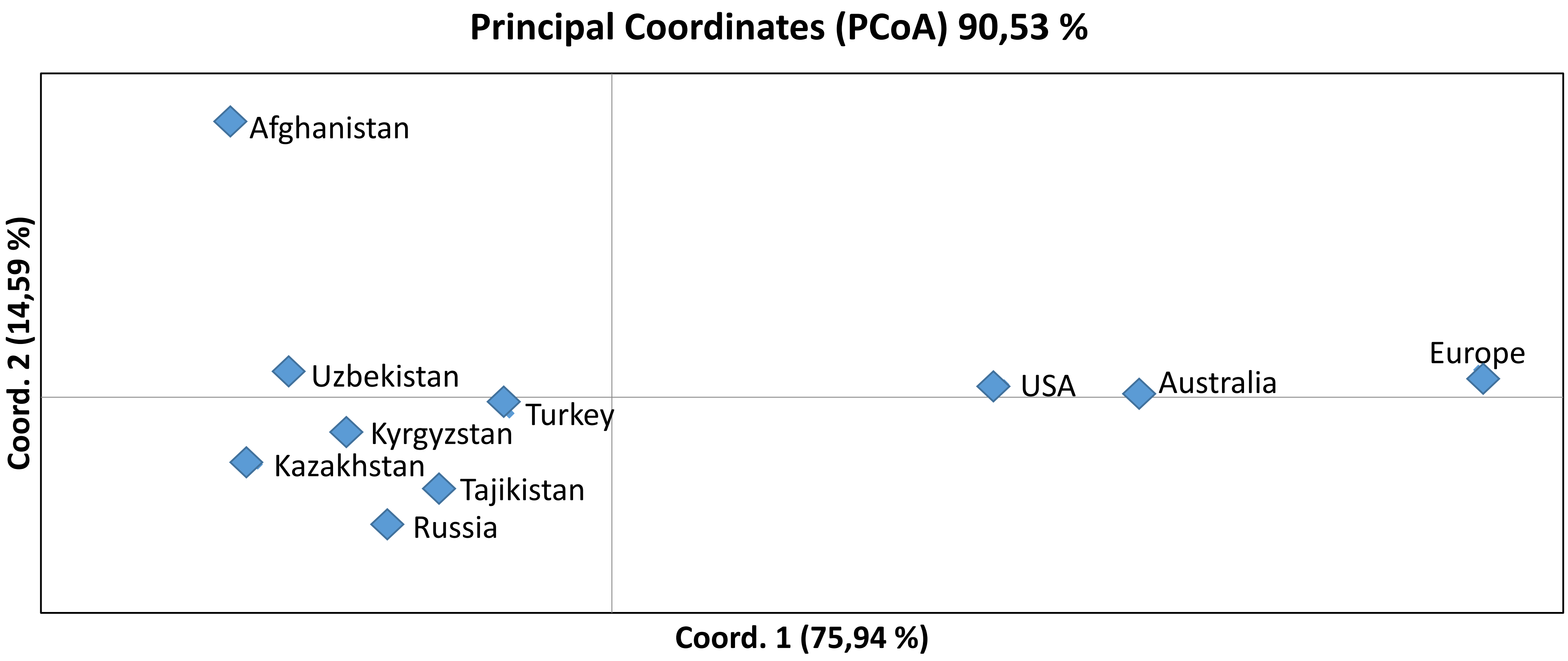

5D chromosome
667 samples
(10 groups)
242 SNPs

Principal Coordinates Analysis (PCoA)

Pairwise Population Matrix of **Nei Unbiased Genetic Distance**

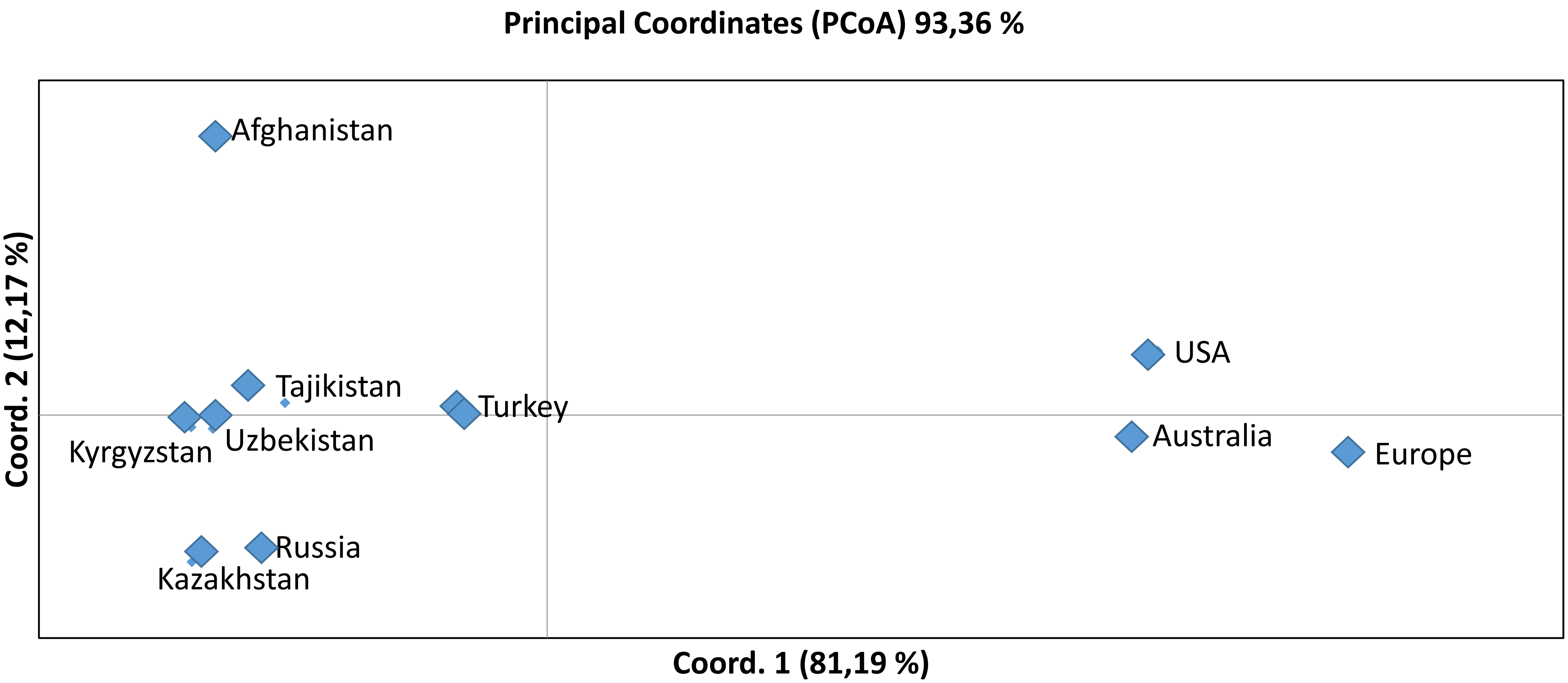

6A chromosome
667 samples
(10 groups)
558 SNPs

Principal Coordinates Analysis (PCoA)

Pairwise Population Matrix of **Nei Unbiased Genetic Distance**

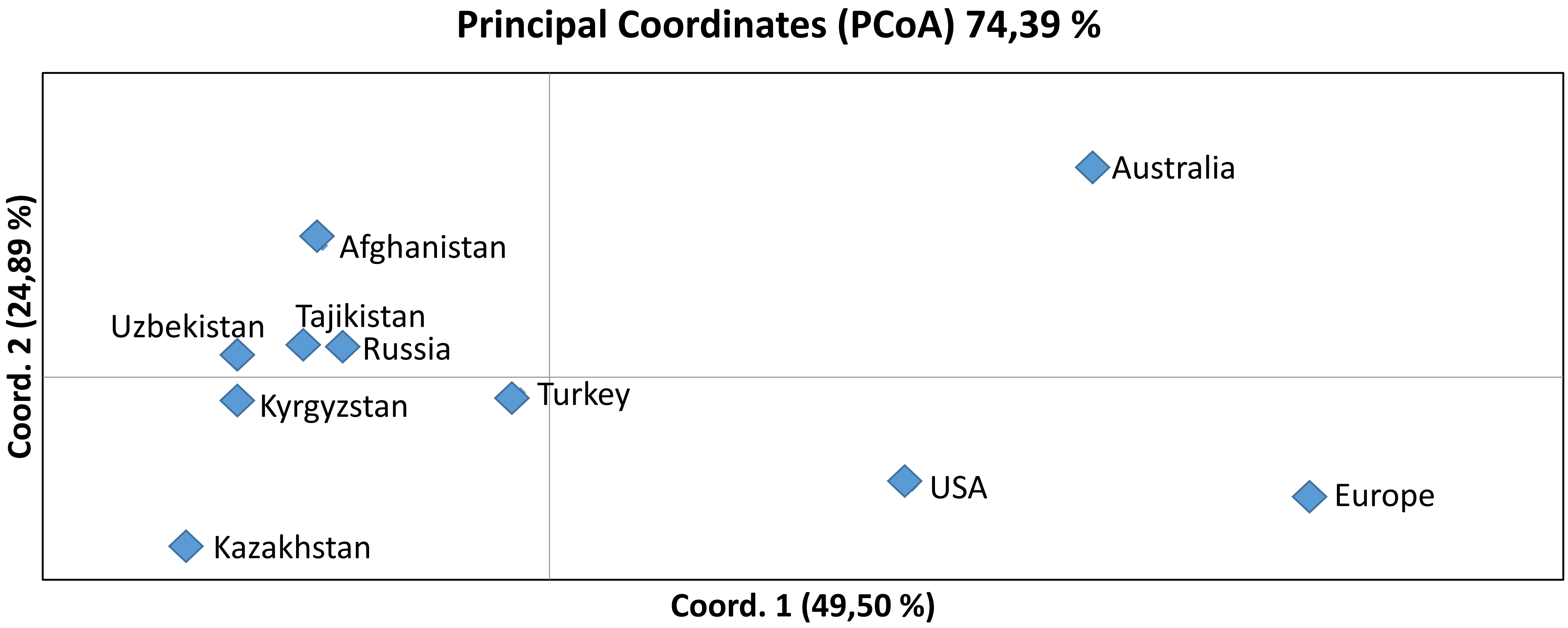

6B chromosome
667 samples
(10 groups)
715 SNPs

Principal Coordinates Analysis (PCoA)

Pairwise Population Matrix of **Nei Unbiased Genetic Distance**

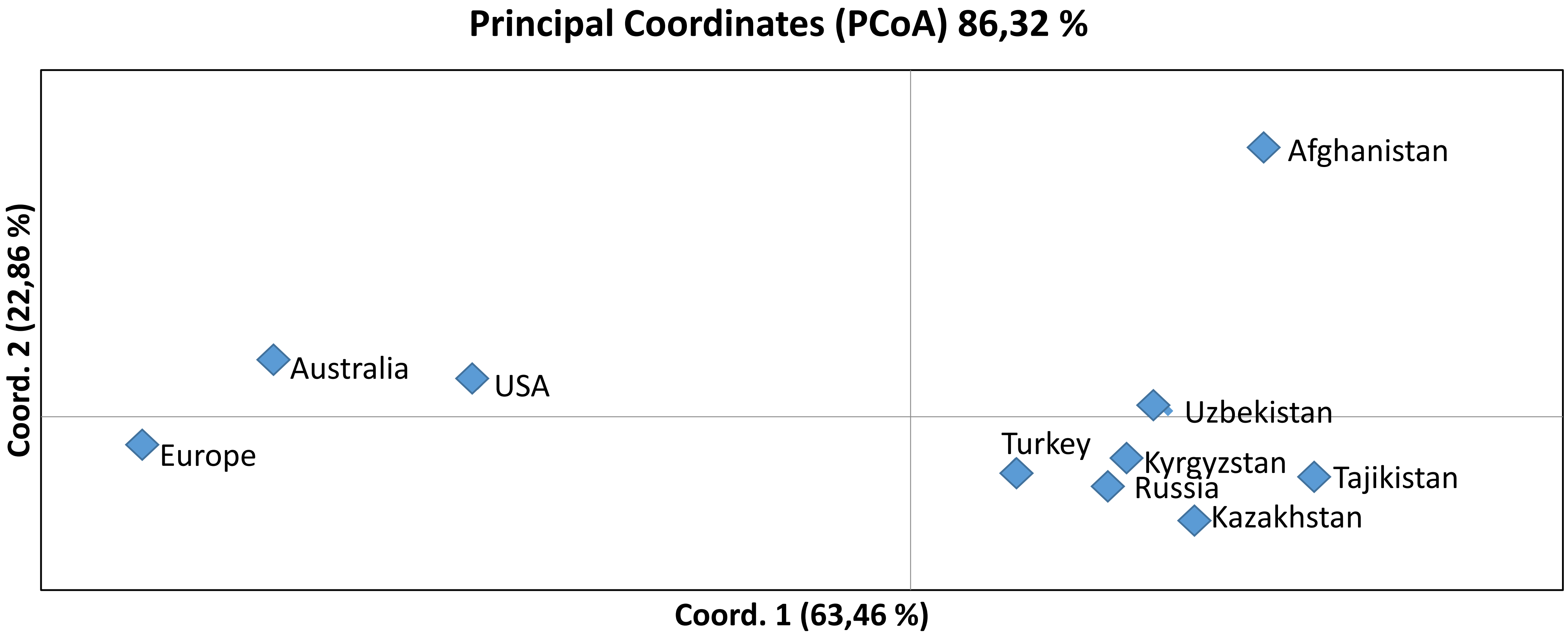

6D chromosome
667 samples
(10 groups)
115 SNPs

Principal Coordinates Analysis (PCoA)

Pairwise Population Matrix of **Nei Unbiased Genetic Distance**

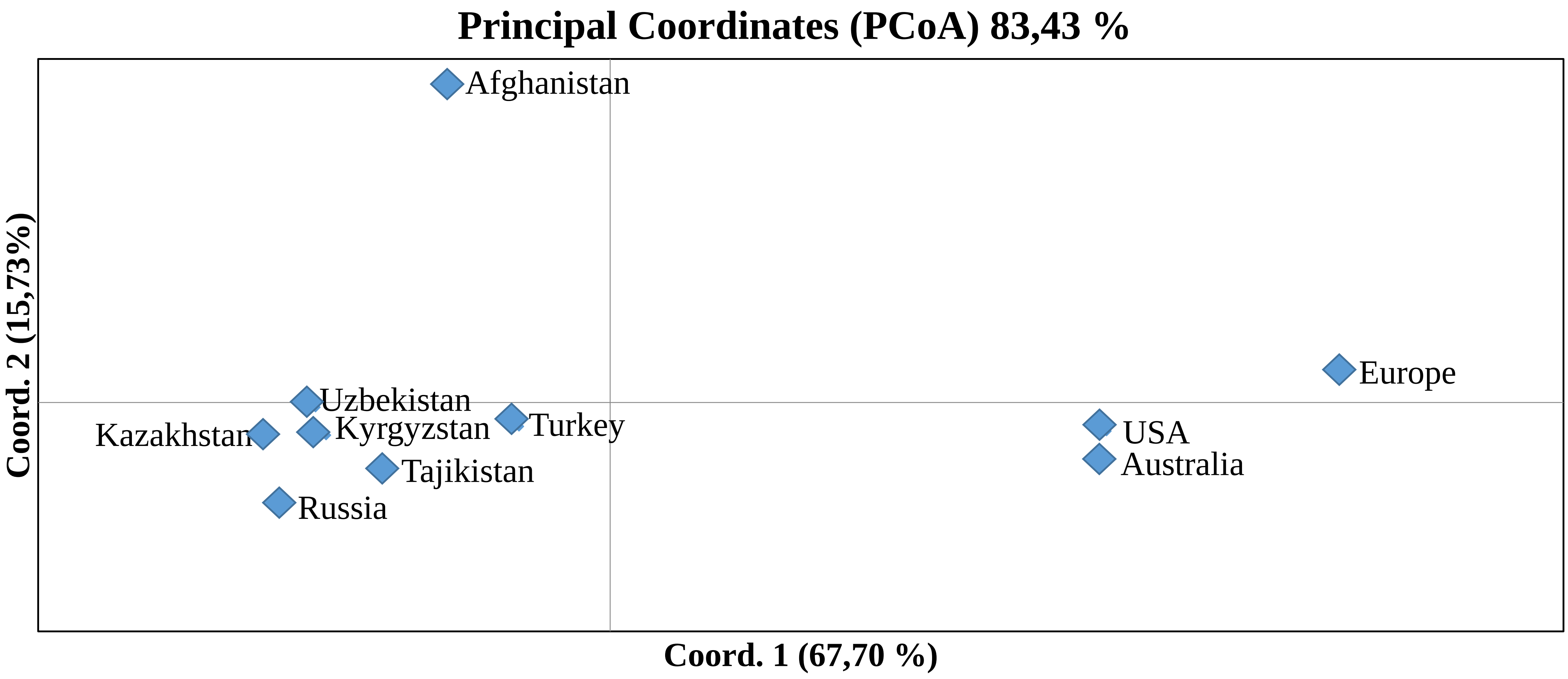

7A chromosome
667 samples
(10 groups)
639 SNPs

Principal Coordinates Analysis (PCoA)

Pairwise Population Matrix of **Nei Unbiased Genetic Distance**

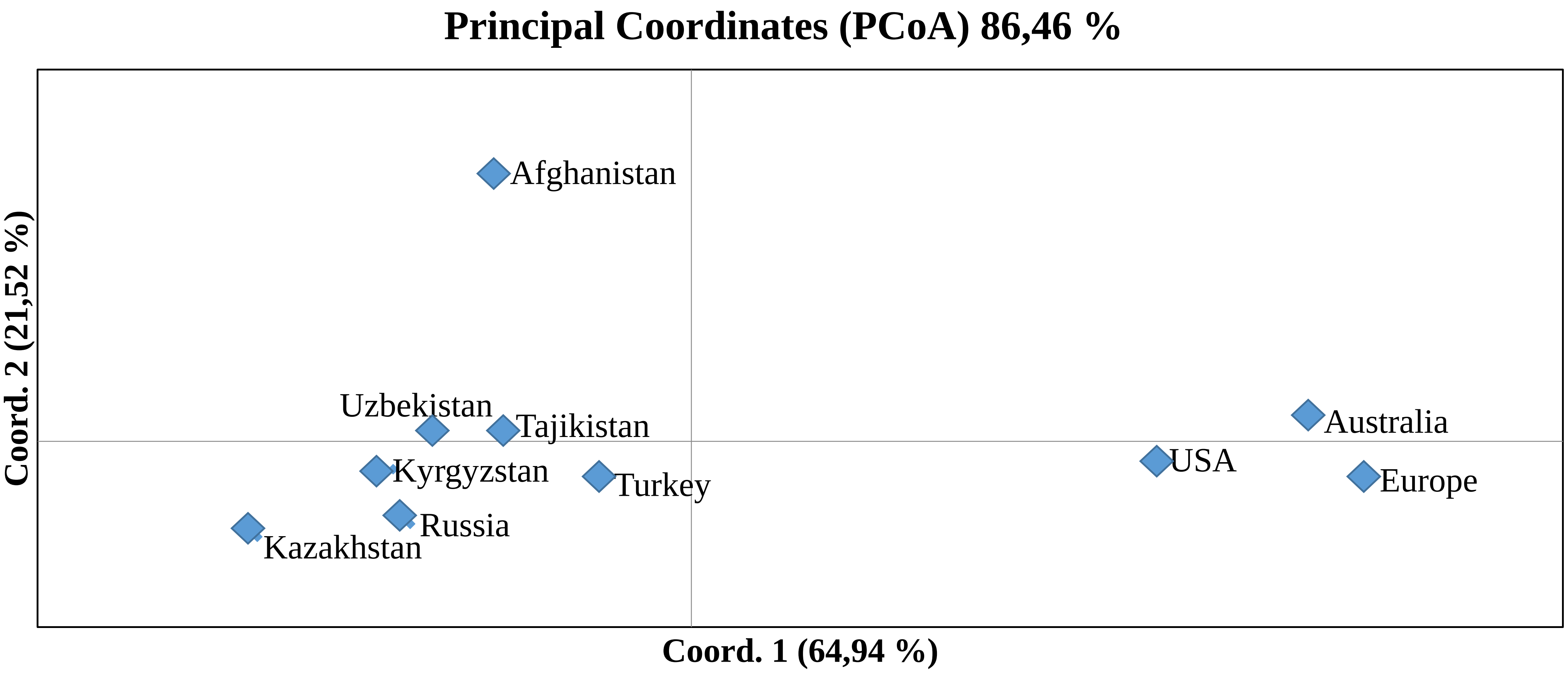

7B chromosome
667 samples
(10 groups)
495 SNPs

Principal Coordinates Analysis (PCoA)

Pairwise Population Matrix of **Nei Unbiased Genetic Distance**

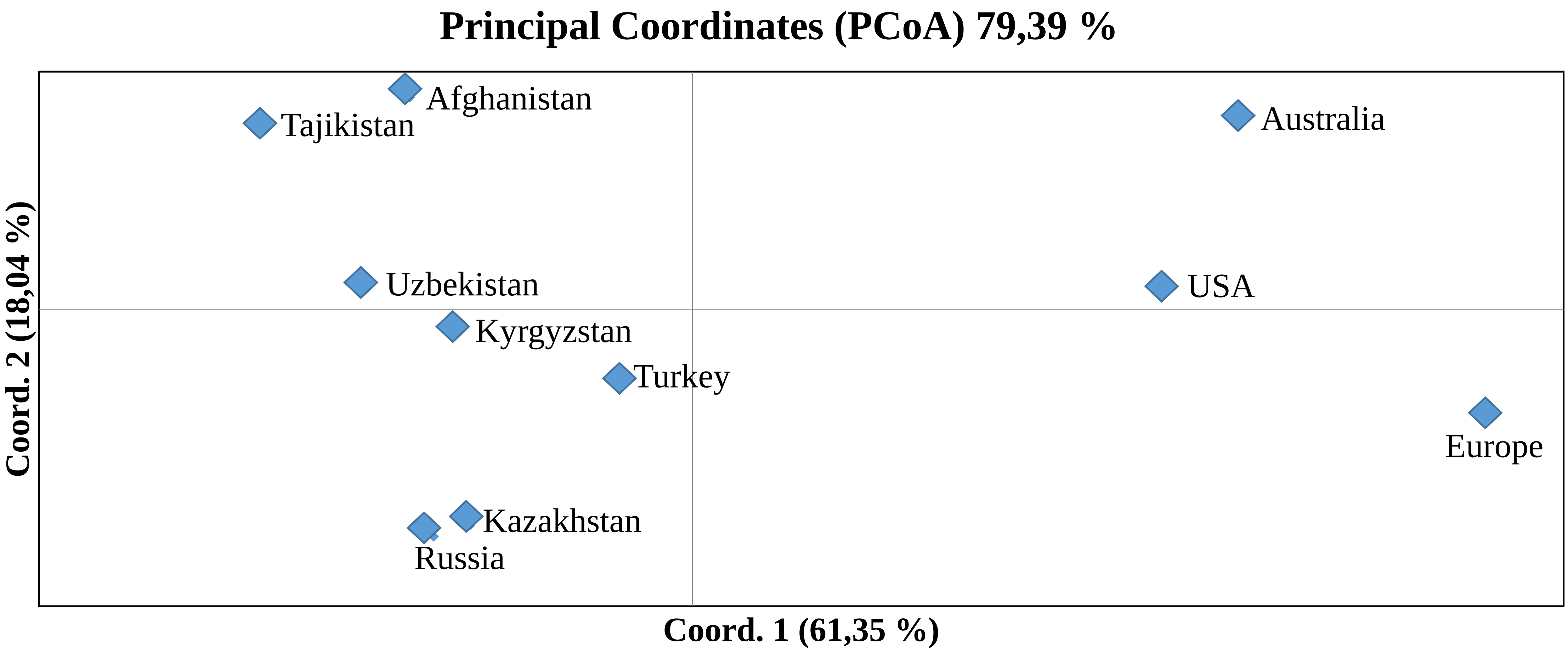

7D chromosome
667 samples
(10 groups)
186 SNPs

Principal Coordinates Analysis (PCoA)

Pairwise Population Matrix of **Nei Unbiased Genetic Distance**

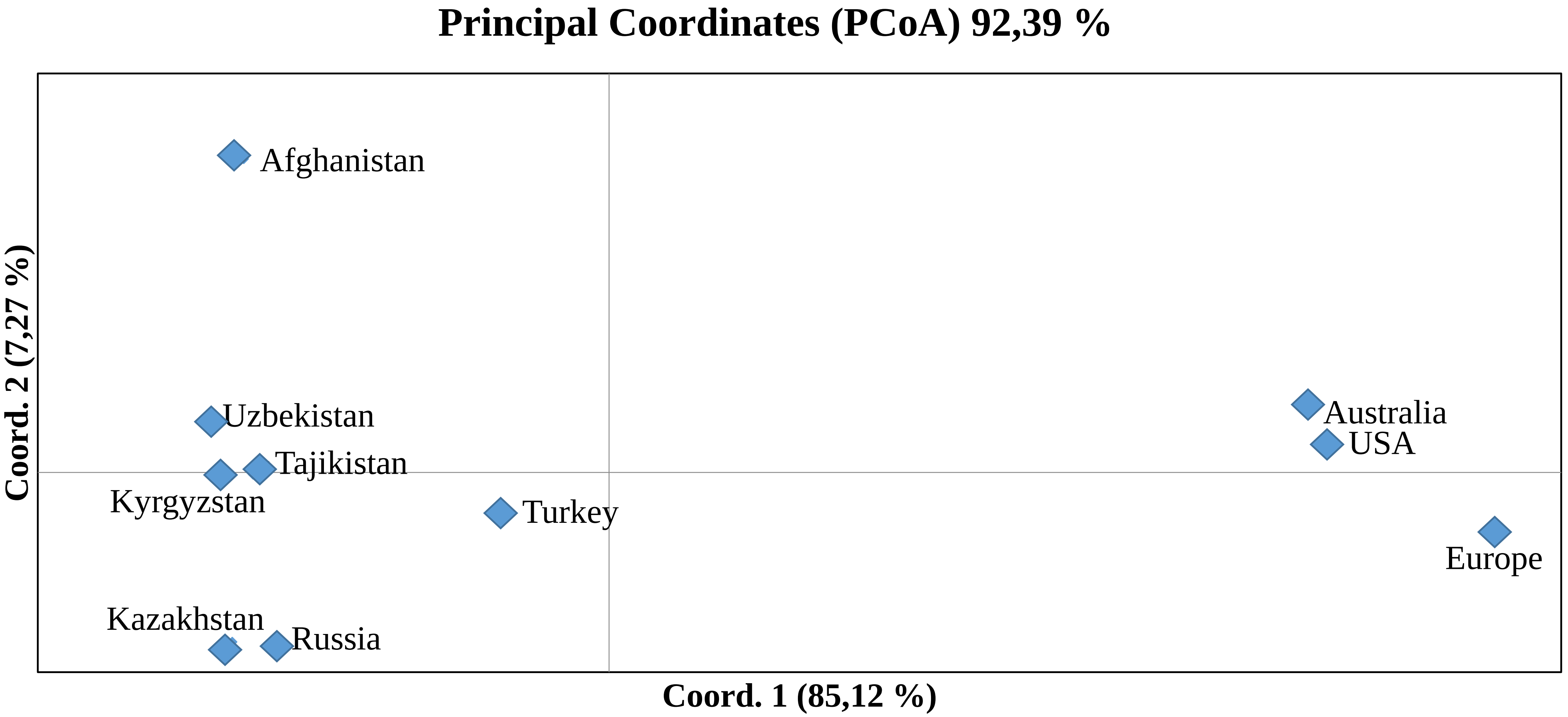

Supplement: Supplementary file 1 [file plants-12-02233-s001.zip › Figure S1.pdf]
